# Thymoquinone in Ocular Neurodegeneration: Modulation of Pathological Mechanisms via Multiple Pathways

**DOI:** 10.3389/fncel.2022.786926

**Published:** 2022-03-02

**Authors:** Nur Musfirah Mahmud, Luminita Paraoan, Nurliza Khaliddin, Tengku Ain Kamalden

**Affiliations:** ^1^UM Eye Research Centre, Department of Ophthalmology, University of Malaya, Kuala Lumpur, Malaysia; ^2^Department of Eye and Vision Science, Institute of Life Course and Medical Sciences, University of Liverpool, Liverpool, United Kingdom

**Keywords:** thymoquinone, neuroprotection, retinal pigment epithelium, oxidative stress, age-related macular degeneration

## Abstract

Thymoquinone is a naturally occurring compound and is the major component of *Nigella sativa*, also known as black seed or black cumin. For centuries thymoquinone has been used especially in the Middle East traditionally to treat wounds, asthma, allergies, fever, headache, cough, hypertension, and diabetes. Studies have suggested beneficial effects of thymoquinone to be attributed to its antioxidant, antibacterial, anti-oxidative stress, anti-inflammatory, and neuroprotective properties. Recently, there has been a surge of interest in thymoquinone as a treatment for neurodegeneration in the brain, such as that seen in Alzheimer’s (AD) and Parkinson’s diseases (PD). *In vitro* and *in vivo* studies on animal models of AD and PD suggest the main neuroprotective mechanisms are based on the anti-inflammatory and anti-oxidative properties of thymoquinone. Neurodegenerative conditions of the eye, such as Age-related Macular Degeneration (AMD) and glaucoma share at least in part similar mechanisms of neuronal cell death with those occurring in AD and PD. This review aims to summarize and critically analyze the evidence to date of the effects and potential neuroprotective actions of thymoquinone in the eye and ocular neurodegenerations.

## Introduction

Thymoquinone is the main extract present in *Nigella sativa*, a traditional remedy widely used in Middle East countries. Recent growing evidence has shown antioxidant, anti-inflammatory, anti-bacterial, anti-fungal, and anti-neurodegenerative properties of thymoquinone in various models (Sedaghat et al., 2014; Parlar and Arslan, 2020; Pop et al., 2020).

Neurodegeneration is associated with progressive deterioration of neuronal structures and functions. In the eye the process of neurodegeneration is implicated in several degenerative diseases of the retina. These include glaucoma, age-related macular degeneration (AMD), diabetic retinopathy (DR) and inherited retinal disorders which cause loss of vision (Jadeja and Martin, 2021). Over the years, increasing cases of vision loss has been identified along with increasing age and life span. According to statistical data from World Health Organization (WHO) from 2010, it is estimated that out of 285 million visually impaired people worldwide, 39 million of them were blind, of which 82% were over 50 years old, and 246 million presented low vision. (Pascolini and Mariotti, 2012). Currently, 43.3 million individuals are estimated to be blind, with this number being predicted to increase to 61 million by 2050 (Bourne et al., 2020, 2021). This data highlights the global problem of blindness faced around the world, especially in countries with longer life span and improved healthcare.

Oxidative stress and inflammation are among the main factors associated with neurodegeneration of the retina (Domènech and Marfany, 2020; Jadeja and Martin, 2021). With aging, the metabolic function in the retina is impaired leading to increase in free radicals such as reactive oxygen species (ROS) and reactive nitrogen species (Domènech and Marfany, 2020). Accumulation of ROS leads to various downstream effects including increase in oxidative stress, accumulation of advanced glycation end products, impaired mitochondrial function and changes in signaling pathways which lead to inflammation, cell death, and apoptosis (Giacco and Brownlee, 2010).

Overall, current treatment options of ocular neurodegeneration are focused only at treating the symptoms and delaying the progression of the disease. To date, there is no cure for these diseases. By altering the signaling pathways related to oxidative stress and inflammatory processes, neuronal cell death processes may be delayed. Alternative and newer treatment options, including from plant origins are being explored to increase the available armamentarium of treatment modalities to delay and prevent loss of vision. Thymoquinone is one of potential therapeutic agent to be used in treating eye diseases.

Pubmed database searches on thymoquinone resulted in 1190 research reports from 1960 to October 2020. The first few thymoquinone studies were designed to investigate its effects in relation to allergic reaction (Coenraads et al., 1975; Chakravarty, 1993). In subsequent studies, thymoquinone was shown to exert protective effects against histamine release by decreasing intracellular calcium uptake, inhibiting protein kinase C and inhibition of oxidative energy metabolism, contributing to some inhibition of histamine release. Interestingly, there were 651 study reports made available from the last 5 years, showing a strongly increasing interest in this compound. Recent reports were further focused on the effect of thymoquinone with anti-inflammatory, antioxidant, and anti-cancer properties. Given the growing evidence of its effect on inflammatory and oxidative stress pathways, thymoquinone emerges as a compound with therapeutic potential for managing ocular neurodegenerative diseases.

## Pharmacological Properties of Thymoquinone

**FIGURE 1:**
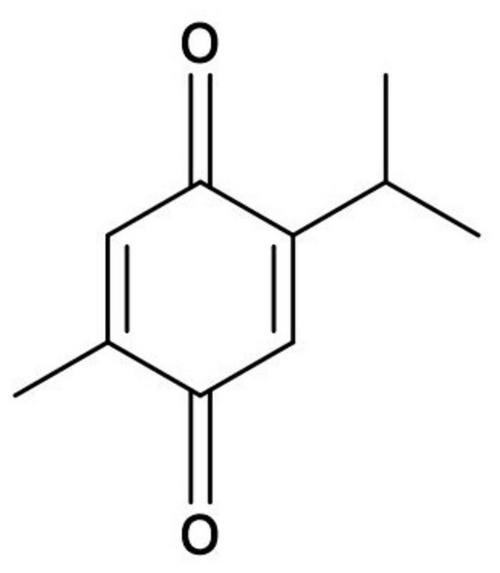
Chemical structure of thymoquinone.

Despite the advancement of medical management, herbal-based medicine is still being practiced in certain parts of the world. The wide utilization of herbal drugs as traditional remedies has encouraged scientists to investigate their active ingredients and their effects on health. Black seed or *Nigella sativa* is widely used as traditional medicine in the Middle East and North America (Badary et al., 2003). Thymoquinone (chemical name 2-isopropyl-5methyl-1,4-benzoquinone; Figure 1; Silachev et al., 2015) is the active compound with the most pharmacological properties isolated from *Nigella sativa*. Its molecular formula is C_10_H_12_O_2_ and has a molar mass 164.20 g/mol. Thymoquinone is a hydrophobic molecule with solubility in 0.1N hydrochloric acid and PBS at different pH levels ranging from 5 to 9. Thymoquinone is shown to be more stable at a lower pH and its stability will decrease with increasing pH. In addition, thymoquinone is found to be light and heat sensitive, which contributes to its short half-life (Goyal et al., 2017).

The protective effect and therapeutic values of thymoquinone have been documented for various conditions such as asthma (Ammar el et al., 2011; Kalemci et al., 2013; Keyhanmanesh et al., 2014), wound healing (Selçuk et al., 2013; Siriwattanasatorn et al., 2020), liver damage (Zeinvand-Lorestani et al., 2018; Atteya et al., 2019), diabetes (El-Shemi et al., 2018; Rani et al., 2018), degenerative diseases (Angeles et al., 2016; Ebrahimi et al., 2017; Elibol et al., 2020), and inflammation-related diseases (Pop et al., 2020). Thymoquinone has been recognized to exert anti-inflammatory and antioxidant properties (Parlar and Arslan, 2020; Savran et al., 2020). However, the main concern in using thymoquinone is its delivery system. Thymoquinone is an oil-based compound with poor oral bioavailability and low solubility. Currently, two lipid-based drug delivery system has been used to improve the solubility and bioavailability of thymoquinone namely thymoquinone-solid-lipid nanostructure (TQ-SLN) and thymoquinone- nanostructured lipid carrier (TQ-NLC). Between these two, TQ-NLC was shown to provide a better and sustained drug release pattern which is a better option to maintain drug concentration for therapeutic approach. TQ-NLC is designed to be 50nm in size with improved encapsulation efficiency and can be stable for up to 24 months. Comparison on the efficacy of oral and intravenous administration of TQ-NLC in terms of thymoquinone delivery and distribution in rat organs indicate that absorption is higher when administered intravenously compared to orally. However, in terms of relative bioavailability, the oral route is superior (Zakarial Ansar et al., 2020).

## Thymoquinone Studies on Neurodegeneration

The increasing number of studies on thymoquinone’s effect on various conditions and diseases demonstrate the compound’s significance as a therapeutic target, which is primarily mediated through changes in signaling pathways related to ROS scavenging and antioxidant production in inflammatory and degenerative diseases (Parlar and Arslan, 2020; Tabeshpour et al., 2020; Tian et al., 2020; TÜrkÖz et al., 2020). Recently, there has been a surge of interest in thymoquinone as a treatment for neurodegeneration in the brain, such as that seen in Alzheimer’s disease (AD) and Parkinson’s disease (PD) (Ebrahimi et al., 2017; Elibol et al., 2020). Both AD and PD share disease mechanisms, owing to an increase in oxidative stress and inflammatory activation which occur with aging and these two factors also play an important role in AMD.

Alzheimer’s disease is a neurodegenerative disorder characterized by accumulation of amyloid beta in the hippocampus. PD is a disease caused by degeneration of dopaminergic neurons in a specific area of the brain called substantia nigra. Several *in vitro* and *in vivo* neuroprotective studies have been conducted on various cell lines and animal models of AD and PD (Table 1). Thymoquinone dose ranging from 100 nm to 12.5 μM were used in *in vitro* neurodegenerative study models (Radad et al., 2009; Khan et al., 2012; Alhebshi et al., 2013, 2014; Ismail et al., 2013; Angeles et al., 2016; Cobourne-Duval et al., 2016, 2018; Alhibshi et al., 2019; Dong et al., 2020). In animal studies, different doses of thymoquinone were administered either by intraperitonial, intravenous or intragastric routes. The experimental studies’ dosing of thymoquinone were between 10 mg/kg and 500 mg/kg, however 10 mg/kg and 20 mg/kg were the most common concentrations used (Ibrahim AbdEl Fattah et al., 2016; Ebrahimi et al., 2017; Ismail et al., 2017a,b; Abulfadl et al., 2018b,c; Dalli et al., 2018; Poorgholam et al., 2018; Ardah et al., 2019; Elibol et al., 2020). The results of these studies in AD and PD clearly showed that thymoquinone could ameliorate the factors that lead to neurodegeneration suggesting that thymoquinone exhibits neuroprotective activities.

**TABLE 1:** *In vitro* and *In vivo* studies on the neuroprotective effects of thymoquinone in AD and PD models.

Model	Cell line/animal model	Thymoquinone dosage	Results	References
AD	Adult female Sprague Dawley rats	10 mg/kg or 20 mg/kg intragastrically for 15 days	In A1–42 infusion rats, thymoquinone treatment eliminated A plaques, restored neuron viability, reduced fibril deposition, mir29c and Bax gene expression, as well as decreased phosphorylated-tau and BACE-1 protein expression.	Elibol et al., 2020
	Human induced pluripotent stem cell (hiPSC)-derived cholinergic neurons	100 nM	Thymoquinone protected hiPSC from cell death and apoptosis caused by Aβ1–42 by increased the glutathione levels in the intracellular antioxidant enzyme, inhibited the generation of ROS, and decreased synaptic toxicity caused by Aβ1–42.	Alhibshi et al., 2019
	Male Wistar rats	5 and 10 mg/kg thymoquinone on a daily basis for 4 weeks.	Thymoquinone reduced the development of amyloid beta plaques in the CA1 region of the hippocampus, increased latency time, and increased the number of surviving neurons.	Poorgholam et al., 2018
	BV-2 microglial cells	12.5 μM for 24 h	In LPS/IFN-activated BV-2 microglial cells, thymoquinone increased expression of neuroprotective proteins (glutaredoxin-3, biliverdin reductase A, 3-mercaptopyruvate sulfotransferase, mitochondrial lon protease) while decreasing pro-inflammatory (IL-2, IL-4, IL-6, IL-10, IL-17) and NF-kB related target genes (IL6, CFB, C3, CXCL3, CCL5).	Cobourne-Duval et al., 2018
	Sprague Dawley male albino rats	20 mg/kg/day intraperitonially for 14 days.	Thymoquinone significantly improved cognition, increased SOD and TAC, decreased AChE activities, decreased MDA and NO levels, reduced TNF-α immunoreactivity and increased BDNF and Bcl-2 levels as well as ACh immunoreactivity.	Abulfadl et al., 2018a
	Sprague-Dawley male albino rats	10, 20, and 40 mg/kg/day orally for 14 days	Thymoquinone increased cognitive impairment in AD rats and reduced Aβ formation. Thymoquinone also significantly reduced TNF- α and IL-1β as well as downregulating the expression of TLRs pathway key effectors and downstream effectors NF-κB and IRF-3 mRNAs at all dose levels.	Abulfadl et al., 2018b
	Adult female Sprague Dawley rats	20 mg/kg/day in corn oil, intragastrically for 15 days	Thymoquinone activated JNK protein, downregulated phosphorylated Tau protein, upregulate dmir-124, and downregulated of ERK1/2 and NOS enzymes.	Dalli et al., 2018
	Male Sprague Dawley rats	20–500 mg/kg BW oral gavage daily for 3 months	Thymoquinone-rich fraction nanoemulsion (TQRFNE) decreased Aβ40 and Aβ42 levels by modulating APP processing, up-regulating IDE and LRP1, and down-regulating BACE1 and RAGE in response to high fat/cholesterol diet-induced rats	Ismail et al., 2017a
	Male Sprague Dawley rats	20–500 mg/kg oral gavage daily for 3 months	Thymoquinone-rich fraction nanoemulsion (TQRFNE) improved memory impairments, increased lipid peroxidation, soluble AB1–40 and AB1–42 levels, as well as increased antioxidants genes expression levels.	Ismail et al., 2017b
	BV-2 microglia	12.5 μM	Thymoquinone decreased superoxide, nitric oxide production, levels lipid hydroperoxides, superoxide dismutase, catalase, and increased level of glutathione in LPS/IFNγ-activated microglia and H_2_O_2_-activated microglia.	Cobourne-Duval et al., 2016
	Male albino rats	10 mg/kg by intraperitonially for 5 days	Thymoquinone reduced acidophilic masses, deformed neurons, and activated glial cells in the frontal lobe of LPS stimulated AD rat model.	Ibrahim AbdEl Fattah et al., 2016
	Primary cultured cerebellar granule neurons (CGNs)	0.1 and 1 μM	Thymoquinone protected CGN from AB1–40 by increasing viability, preserving CGN’s intact cell bodies and neurite network, lowering free radical development, and reducing caspase-3, -8, and -9 activation.	Ismail et al., 2013
	E18	100 nM	Thymoquinone enhanced cells survival, improved mitochondrial function, reduced intracellular ROS, reduced AB-induced inhibition of synaptic vesicle recycling, and inhibited Ab1–42 aggregation.	Alhebshi et al., 2013
	PC-12	0.78–400 μM	Thymoquinone increased cell viability and mitochondrial membrane potential (MMP), decreased TBARS content, nitric oxide (NO) and activity of acetylcholine esterase (AChE), and improved glutathione and its dependent enzymes (glutathione peroxidase, glutathione reductase) in Aβ25–35 treated PC 12 cells.	Khan et al., 2012
PD	SH-SY5Y and C57/BL6 mice	*In vitro*: 0.25, 0.5, and 0.75 μM *In vivo*: 10 mg/kg BW daily for 7 days	Thymoquinone inhibited MPP + -induced cell death and apoptosis *in vitro* by elevating nuclear translocation of Nrf2 and subsequently the expression of antioxidative genes such as Heme oxygenase 1 (HO-1), quinone oxidoreductase (NQO1) and Glutathione-S-Transferase (GST) *in vitro*. Thymoquinone *in vivo* administration ameliorated oxidative stress and effectively mitigated nigrostriatal dopaminergic degeneration by activating the Nrf2-ARE pathway.	Dong et al., 2020
	Male C57BL/6c mice	10 mg/kg BW intraperitoneally for 7 days	Thymoquinone administration increased activities of superoxide dismutase and catalase, increased glutathione level, reduced malondialdehyde formation, reduced pro-inflammatory cytokines, downregulated inflammatory mediators cyclooxygenase-2 (COX-2) and inducible nitric oxide synthase (iNOS) as well as restored dopamine neuron loss in the striatum following MPTP administration.	Ardah et al., 2019
	Male Wistar rats	7.5 and 15 mg/kg/day	TQ prevented rotenone-induced motor defects and changes in the Parkin, Drp1, dopamine and tyrosine hydroxylase levels.	Ebrahimi et al., 2017
	SK-N-SH and primary cortical neuronal lines	673 nmol/L	Thymoquinone reduced loss in dopaminergic neurons, oxidative dysfunction, and locomotor defects in mutated leucine-rich repeat kinase-2 neuronal cells.	Angeles et al., 2016
	Adult male unilateral 6-OHDA-lesioned Wistar rats	10 mg/kg po three times	Thymoquinone pre-treatment significantly improved turning behavior, prevented loss of SNC neurons, and lowered level of MDA caused by apomorphine.	Sedaghat et al., 2014
	E18 and human iPSC (hiPSC)-derived neurons	100 nM	Thymoquinone reduced loss of synaptophysin, enhanced synaptic vesicle recycling, protected against synapse damage, maintained firing activity and inhibited synaptic activity in αSN-induced E18 and hiPSC cells.	Alhebshi et al., 2014
	Primary dopaminergic cell	0.01, 0.1, 1, and 10 μM	Thymoquinone protected dopaminergic neuronal cell death from rotenone and MPP + toxicity.	Radad et al., 2009

Taken together, these studies showed that thymoquinone has a neuroprotective effect mediated via modulation of oxidative stress, reduced formation of AB plaques, control of mitochondrial integrity and functions, as well as regulation of inflammation-related genes, thus preventing neuronal cell death and apoptosis. These findings suggested that thymoquinone could be an effective neuroprotective agent in treating AD and PD. Due to similar neurodegenerative mechanisms, it was also suggested that thymoquinone could be exploited to study its possible protective effect for ocular neurodegeneration diseases (Kaarniranta et al., 2011).

## Thymoquinone Studies in Other Eye Conditions

There are limited studies on the positive effects of thymoquinone in other eye conditions. Studies of the effect of thymoquinone on the eye focused on dry eyes (Kocatürk et al., 2018), conjunctivitis (Hayat et al., 2011), cataract (Fouad and Alwadani, 2015; Taysi et al., 2015), glaucoma (Fahmy et al., 2018), and oxidative stress in the RPE (Hu et al., 2019). Table 2 summarizes the findings of these studies on various eye conditions.

**TABLE 2:** A summary of studies on thymoquinone effects in various ocular conditions.

Thymoquinone concentration	Experimental model	Study condition	Target tissue	Effects	References
0.05, 0.1, and 0.5% w/v	Mice	Ovalbumin (OVA)-induced allergic conjunctivitis (AC)	Conjunctiva	Thymoquinone attenuated eosinophils recruitment, level of IgE, histamine, and cytokines.	Hayat et al., 2011
20, 40, and 80 mg/kg	Rats	Lens changes in diabetic rats	Lens	Thymoquinone lessen malondialdehyde, nitric oxide, tumor necrosis factor-α, glycated proteins, aldose reductase activity, sorbitol level, caspase-3 activity, glutathione peroxidase, superoxide dismutase, and catalase activities.	Fouad and Alwadani, 2015
50 mg/kg	Rats	Ionizing radiation-induced cataracts	Lens	Thymoquinone decreased cataract formation.	Taysi et al., 2015
N/A	Rabbits	Glaucoma	Aqueous humor	Thymoquinone reduced IOP level.	Fahmy et al., 2018
0.4% w/v	Mice	Dry eye	Conjunctiva	Thymoquinone increased IL-1a and IL-2.	Kocatürk et al., 2018
0, 5, 10, 20, and 40 μM	ARPE-19 cells	Hydrogen peroxide-induced oxidative stress	RPE	Thymoquinone improved cell viability, reduced the levels of ROS, malondialdehyde, bcl-2, increased of bax, cleaved caspase-3, glutathione (GSH) level and superoxide dismutase activity and enhanced the activation of Nrf2/heme oxygenase 1 (HO-1) signaling pathway in H_2_O_2_-induced ARPE cells.	Hu et al., 2019

With regards to allergic conjunctivitis, Hayat et al. (2011) studied the effect of thymoquinone on a mice model of ovalbumin (OVA)-induced allergic conjunctivitis by looking into eosinophil recruitment, IgE levels, histamine and cytokines releases (Hayat et al., 2011). OVA administration caused increase in IgE and OVA-specific immunoglobulin E (IgE) levels, eosinophils recruitment, histamine level, mRNA expressions and protein level of pro-inflammatory cytokines as compared to control groups and thymoquinone was shown to ameliorate this effect. Thymoquinone concentration as low as 0.05% w/v was able to attenuate eosinophil recruitment to the site of allergy. However, at a concentration of 0.5%, thymoquinone showed convincing suppression of ocular symptoms, inflammatory cell infiltration in the conjunctiva, blood and OLF, increased levels of serum and OVA-specific IgE, and histamine levels in OVA-exposed mice. In addition, administration of thymoquinone markedly reduced mRNA expression and serum level of interleukins including 1L-4, IL-5, IL-13 in mice exposed to OVA. In another study, Fouad and Alwadani (2015) investigated the protective effects of thymoquinone against lens changes in streptozotocin-induced diabetic rats (Fouad and Alwadani, 2015). Thymoquinone (20, 40, and 80 mg/kg/day) was administered for 12 weeks. At 40 and 80 mg/kg concentrations, there was significant decrease of many pro-inflammatory factors such as malondialdehyde, and tumor necrosis factor-α, glutathione peroxidase, superoxide dismutase (SOD), and catalase activities. Decreased levels of glycated proteins, aldose reductase activity, sorbitol level, caspase-3 activity, and total and soluble protein contents in the lens were also noted among diabetic rats. These data suggest that thymoquinone protects lens tissue against changes induced by diabetes through its antioxidant, anti-inflammatory, and antidiabetic effects. Kocatürk et al. (2018) studied the effects of thymoquinone on a dry eye model. They showed that thymoquinone was able to cause inflammation observed through pathological examination, but no significant changes were observed in the level of cytokines.

Next, Taysi et al. (2015) studied the antioxidant and radioprotective effects of thymoquinone against ionizing radiation-induced cataracts in the lens of rats exposed to total cranial irradiation (IR) with a single dose of 5 gray (Gy) (Taysi et al., 2015). Thymoquinone (50 mg/kg/day) reduced cataract formation rate from 80 to 50% compared to control. Nitric oxide synthase activity, nitric oxide and peroxynitrite levels were also reduced with thymoquinone administration. The findings of this study indicated that thymoquinone may be a potential compound for preventing cataract formation in IR-exposed lenses by inhibiting the production of oxidative stress.

In the most recent study, Xu et al. (2018) examined the effect of thymoquinone against hydrogen peroxide (H_2_O_2_) -induced oxidative stress in human RPE cells (Xu et al., 2018). Their results showed that thymoquinone (5, 10, 20, and 40 μM) improved cell viability and reduced apoptosis in H_2_O_2_- induced ARPE cells. They also found that the levels of ROS, malondialdehyde, glutathione and SOD activity induced by H_2_O_2_ decreased following thymoquinone pretreatment. The changes caused by thymoquinone were thought to be through Nrf2/Ho-1 signaling pathway as thymoquinone was shown to enhance the activation of Nrf2/heme oxygenase 1 (HO-1) pathway in H_2_O_2_-induced ARPE cells. To support their hypothesis, they showed that this thymoquinone effect was absent in Nrf2-knockout mice model.

These data mentioned above can be a preliminary data that shows the possible protective ability of thymoquinone in eye-related problems. Although, these studies are more focus toward the protective effect of thymoquinone on the disease focusing on the frontal area of the eye, based on evidence of thymoquinone on neurodegenerative disease discussed earlier, it is possible that thymoquinone can be exploited to be a new treatment for neurodegeneration in the eye.

## Effects of Thymoquinone on Signaling Mechanisms of Ocular Neurodegeneration

The effect of thymoquinone on AD- and PD-related neurodegeneration warrants further studies investigating the direct protective effects of thymoquinone in ocular neurodegenerative diseases. The neuroprotective effect of thymoquinone might be cell-specific, thus the effect seen in AD and PD might differ from ocular degeneration models. However, it is important to note that the neuroprotective mechanisms in AD and PD are shown to occur via the anti-inflammatory and anti-oxidative properties of thymoquinone (Sedaghat et al., 2014; Elibol et al., 2019). These include modulation of oxidative stress, anti-inflammatory activity, apoptosis and involve regulation of mitochondria function, NF-kB and Nrf2 signaling pathways and restoration of redox homeostasis (Alhebshi et al., 2013; Ardah et al., 2019).

Intake of antioxidants such as Vitamin C, E, beta-carotene, lutein, and zeaxanthin have been shown to prevent neurodegeneration, thus preserving vision (Payne et al., 2014). Thymoquinone is a naturally occuring plant-based antioxidant and its consumption may potentially provide similar neuroprotective effects like Vitamin C. However further studies into improving delivery methods are required to optimize thymoquinone absorption. For instance, comparison between two delivery routes (intranasal and intravenous route) of 5 mg/kg/day thymoquinone loaded with PLGA-chitosan nanoparticles showed that the intranasal route was better at facilitating the delivery of thymoquinone to the brain (Xiao et al., 2016). Data showed a concentration of 996.43 ± 119.36 ng/mL was delivered to the brain intranasally compared to intravenous at 390.61 ± 54.44 ng/mL. Apart from a better concentration, intranasal route also provides a longer half-life (118.23 ± 3.97 h) and lower elimination rate of thymoquinone. Hence, this study may be a steppingstone in investigating the delivery system of thymoquinone in the case of ocular diseases.

### Effect on Redox Homeostasis

Redox homeostasis is defined as the endogenous ability of the cells in dealing with challenges from various stressors generated by metabolic activities or any exogenous sources. Persistent exposure to the stressor and inadequate ability of the cells to restore the balance leads to oxidative stress formation. Hence, restoration of the redox homeostasis is essential to prevent oxidative stress conditions. For this purpose, the cells increase production of oxidants that acts as signaling species resulting in balance equilibrium in production of pro-inflammatory reaction and pro-oxidant enzymes. The whole process is only aimed to diminish the challenge and restore the homeostasis (Ursini et al., 2016).

In the eye, ROS are not only generated endogenously due to robust metabolic activities. Intense exposure to light, and high atmospheric oxygen exposure also contributed to generation of ROS in the eye (Beatty et al., 2000; Tokarz et al., 2013). Neutralization of this abundantly generated ROS in the eye is crucial for cellular homeostasis. At a low and moderate level, the ROS acts a signaling molecule that sustain cellular proliferation and differentiation as well as activate stress-responsive survival pathways by increasing the expression of pro-oxidant related genes. Persistent exposure to ROS would implicated to various eye diseases such as cataract (Ho et al., 2010), glaucoma (Izzotti et al., 2006), and AMD (Beatty et al., 2000). GSH, NADH, and NADPH are examples of the cellular redox presented in the eye mainly generated from mitochondria-related pathways and their level are reduced with aging (Bradshaw, 2019; Pan et al., 2021).

Thymoquinone has antioxidant capacity that has been found to be able to maintain redox homeostasis in the cells. Thymoquinone has been shown to decrease the level of GSH and increase the level of NADH and NAPDH (Sankaranarayanan and Pari, 2011). These molecules serve important roles in biological oxidation-reduction processes, as a cofactor for many enzymes, and a detoxifying and free radical scavenging agent, hence retain the cell homeostasis. GSH is responsible in maintaining the sulfhydryl (–SH) group of enzymes in its reduced state. The sulfhydryl residues of GSH molecules are easily oxidized to form GSH disulfide (GSSG), which is then reduced back to GSH by the reaction with GSH reductase (Aoyama and Nakaki, 2015). Since RNS and ROS may interact with cysteine thiol group to form electrophile containing –SH, GSH is responsible to attack these molecules and retain it at less reactive state (Armutcu et al., 2018). Decrease in GSH level is accompanied with increase in GSSH is observed in oxidative stress related diseases which include AD and PD (Calabrese et al., 2006; Mischley et al., 2016). The redox capacity of cells is determined by the ratio of GSH/GSSG. In an *in vivo* study, decreased level of GSH was observed in the brain tissue treated with sodium nitrite and it was restored with thymoquinone treatment. In addition, thymoquinone treatment also shown to decrease the level of TNF-α and IL1β and increase level of IL-10 in a concentration-dependent manner (Hamdan et al., 2019). Similar reaction was observed in the model of brain ischemia (Al-Majed et al., 2006). Mass spectrometry analysis suggest that thymoquinone react with GSH by rapid binding of the thymoquinone to the cysteine residues of the GSH molecules in the 3-position of benzoquinone ring, hence prevent GSH oxidation to GSSG (Khalife and Lupidi, 2007).

NADH is the reduced form of NAD+ plays important role in mitochondrial metabolism. The NAD+/NADH ratio is a known regulator of various biological processes in the cells including glycolysis, energy metabolism, mitochondrial function, and gene expression (Xiao et al., 2018). The NADH is essential for ATP production and oxidative phosphorylation in the electron transport chain system. Decrease in the NAD+/NADH ratio, reduced cellular NAD + level, and increased NADH have been observed during aging (Braidy et al., 2011). NADPH is responsible for maintaining cells’ redox state and biosynthesis of fatty acids, cholesterol, and deoxynucleotides. NADPH decreased in aging as a result of decreased of its immediate precursor, NADP+. Therapies that increase NAD + levels and NADP + could greatly benefit subjects with aging-related disorders. Study in BV2 microglial cells showed that thymoquinone was able to reduce LPS-induce ROS by increasing the level of NAD+/NADH ratio. This reaction is followed by the attenuation of cellular ROS level (Velagapudi et al., 2017a). In addition, thymoquinone was also showed to play a major role in hyperglycemia model which is important in DR pathogenesis. A significant drop of 45 and 30% of NADH and NADPH was observed with 5 μm of thymoquinone incubation (Gray et al., 2016). The mechanism on how thymoquinone regulate the NAD+, NADH, NADP+, and NADPH is still not clear. Some research suggest it happens through inhibition of p47PHOX, a cytosolic subunit of the NADPH oxidase, phosphorylation on Ser-304 and Ser-328 (Boudiaf et al., 2016).

### Effect of Thymoquinone on Modulation of Oxidative Stress

Oxidative stress has long been linked to aging and neurodegenerative disorders, including those affecting the eyes (Jadeja and Martin, 2021). An imbalance in the accumulation of harmful substances in cells and the cell’s normal ability to avoid oxidative damage by using its anti-oxidative properties is referred to as oxidative stress (Beatty et al., 2000; Burton and Jauniaux, 2011; Zafrilla et al., 2013). Under normal homeostasis, the antioxidant would neutralize the oxidative stress level to ensure cell survival. Persistent stress, on the other hand, will disrupt this protective mechanism, causing cells to undergo apoptosis, necrosis, or autophagic cell death. Oxidative stress is accompanied by the increase in ROS in the cells. The major types of ROS are superoxide anion, hydrogen peroxide, hydroxyl radical, peroxy radical, and the second messenger nitric oxide (Burton and Jauniaux, 2011). A major source of ROS production in cells is the mitochondrial respiratory chain. Oxidative stress accumulation in cells is detrimental because it causes cellular damages to proteins, lipids and nucleic acids (Cui et al., 2012). Excessive accumulation of ROS can trigger downstream signaling pathways such as NF-κB, ERK1/2, p38 MAPK, and autophagy-related signaling (Zhang et al., 2016).

Due to a variety of factors, including high oxygen intake, excessive light exposure, and the presence of polyunsaturated fatty acids, the retina is extremely susceptible to oxidative stress harm. Furthermore, since the outer segments of photoreceptors are constantly shed, the RPE’s phagocytic role adds to the oxidative burden (Beatty et al., 2000; Tokarz et al., 2013). The accumulation of oxidative stress disrupts cell homeostasis and triggers a defensive cell response in photoreceptors and retinal ganglion cells (Nita and Grzybowski, 2016). These two cell types are high-metabolizing neurons that are constantly exposed to oxidative stress insults (Beatty et al., 2000).

Thymoquinone is known to reduce *in vitro* and *in vivo* oxidative stress levels. Various studies showed the ability of thymoquinone to reduce oxidative stressors such as TNFα, lipopolysaccharides, IL-1β and Il-1α levels (Wang D. et al., 2015; Cobourne-Duval et al., 2018). In the case of neurodegenerations, Sedaghat et al. (2014) has shown that thymoquinone was able to ameliorate the oxidative stress marker [malondialdehyde (MDA) and nitrile] levels in unilateral intrastriatal 6-hydroxydopamine (6-OHDA)-lesioned rats as a model of PD (Sedaghat et al., 2014). In another study, thymoquinone significantly decreased MDA, nitrile oxide and TNF-α level in an AD model (Abulfadl et al., 2018b). A similar observation was made in LPS-induced oxidative stress in BV2 microglialcell, where 10um of thymoquinone was able to reduce the inflammatory cytokines expressions through qPCR and ELISA (Cobourne-Duval et al., 2018). In addition, thymoquinone treatment (2.5, 5, and 10 mM) was shown to reduce the levels of TNF-a, IL-6, and IL-1b in BV2 cells challenged by LPS at both mRNA and protein levels (Velagapudi et al., 2017a). Thymoquinone (3, 6, 12 μM) was found to exert its effect by reducing the level of nitrite oxide and PGE2 production indicated in BV2 cells (Wang Y. et al., 2015). On the contrary however, Elibol et al. (2019) showed that in the case of AD thymoquinone did not change the level of TNF-α, IL-1α, and IL-1β groups with pretreatment of thymoquinone (Elibol et al., 2019).

In addition to reducing oxidative stressors, thymoquinone has been shown to increase inherent intracellular antioxidants such as SOD, catalase and glutathione. In an *in vivo* study of arsenic-induced neurotoxicity, 10 mg/kg of thymoquinone suppressed levels of oxidative stressors (NO and TNF-α) and increasing antioxidants (glutathione, glutathione peroxidase, SOD, and catalase) in the rat’s nervous system (Kassab and El-Hennamy, 2017). The same effect was seen in lead (Mabrouk, 2017) and paraquat toxicity (Zeinvand-Lorestani et al., 2018) in rat’s liver. Co-treatment with 5 mg/kg/day of thymoquinone for 5 weeks resulted in increased in reduced glutathione levels, but also SOD, glutathione peroxidase, catalase and glutathione reductase activities (Mabrouk, 2017). Meanwhile, pretreatment with 10 mg/kg thymoquinone was shown to restore SOD activity in paraquat-induced toxicity (Zeinvand-Lorestani et al., 2018). These studies show the ability of thymoquinone in ameliorating the level of antioxidant in hepatotoxicity injury.

The effect of thymoquinone on different cell types may vary and possibly are cell specific. However, it is possible that thymoquinone provide protective effects on the retina by reducing oxidative stress and increasing antioxidant properties. Therefore, it is worthwhile to examine the effects of thymoquinone on ocular neurodegenerative models.

### Anti-inflammatory Effects of Thymoquinone

Inflammatory response occurs as a result of modulation of oxidative stress involving a series of cellular events that allow the body to defense itself from intruders or further damage (Hussain et al., 2016). Accumulation and prolong exposure to oxidative stress triggers exaggerated inflammatory response of cells termed as chronic inflammation. Studies showed that chronic inflammation does play an important role in age-related disease (Sharif et al., 2019). Inflammation is considered as the primary immune system reaction to eliminate pathogens or other stimuli in order to restore the cells to normal state (Chen et al., 2017).

In the eye, there five neuronal cells present namely photoreceptors, bipolar cells, ganglion cells, horizontal cells, and amacrine cells. These cells are responsible in providing vision. With aging, these neuronal cells undergo various physiological and morphological changes due to various endogenous and exogenous factors including oxidative stress and inflammation, which leads to impaired vision. Recently, inflammation has become a concern for AMD, DR and macular edema as a secondary to DR (Das, 2016; Tan et al., 2020). The importance of inflammation in these diseases has been discussed (Ascaso et al., 2014; Kauppinen et al., 2016; Forrester et al., 2020). Apart from anti-VEGF treatment, anti-inflammatory agents such as corticosteroids has been used to delay the progression of these disease (Wang et al., 2011). Macrophages and giant cells have been reported to localize near drusen, at the breakdown of Bruch’s membrane, and in the CNV membrane of AMD. Furthermore, increase in inflammatory cytokines such as tumor necrosis factor-α (TNF-α) and IL-1, was also found in the RPE, thereby inducing additional inflammatory cellular infiltration and accelerates angiogenesis and CNV formation in AMD (Wang et al., 2011). VEGF is a growth factor responsible in promoting angiogenesis. However in DR and AMD, the presence of VEGF cause uncontrolled growth of blood vessels and promote disease progression. Increase of VEGF and its involvement in ocular disease is well studied (Homayouni, 2009; Ahuja et al., 2019).

Microglia activation is considered first line of defense against inflammation followed by release of anti-inflammatory mediators and resolution of the inflammatory response. It is well known that microglia activation is an important in pathogenesis of AD and PD (Qian and Flood, 2008; Mandrekar-Colucci and Landreth, 2010). Analysis on microglia cells exposed to thymoquinone demonstrated a significant anti-inflammatory property of thymoquinone observed through attenuation of IL-6, CCL12/MCP-5, and CCL2/MCP-1 protein and mRNA expression (Taka et al., 2015). In another study, thymoquinone showed anti-inflammatory properties by inhibition of nitric oxide (NO), PGE2, TNF-α, and IL-1β production in BV2 microglial cells (Wang Y. et al., 2015). Besides, thymoquinone anti-inflammatory activity in AD has been observed through alleviation of TLR signaling pathway, a signaling pathway responsible for innate response. Different thymoquinone dosage ranging from 10 mg/kg/day to 40 mg/kg/day was able to reduce TLR-2, TLR-4, MyD88, TRIF and IRF-3, which are related to TLR signaling (Abulfadl et al., 2018a). In conclusion, these findings suggest a possible anti-inflammatory effect of thymoquinone through attenuation of various inflammatory related cytokines, which could provide therapeutic value in neurodegenerative diseases and ocular disease.

### Effects of Thymoquinone on Advanced Glycation Endproducts

Accumulation of oxidative stress are reported to cause peroxidation of polyunsaturated fatty acids in the cell membrane of the brain resulting in the formation of toxic metabolites such as MDA and 4HNE, as well as advanced glycation end products (AGEs). Long-lived proteins and protein deposits in human and animal tissues, including the retina, have been shown to contain these byproduct metabolites. The AGEs is a byproduct of Maillard reaction through interaction of aldehyde or carbonyl sugar with amino group of proteins, lipids or nucleic acids (Goldin et al., 2006; Luevano-Contreras and Chapman-Novakofski, 2010). In the eye specifically, AGEs are associated with age-related macular degeneration (AMD), cataract formation, diabetic retinopathy and glaucoma (Ishibashi et al., 1998; Howes et al., 2004).

Advanced glycation end products accumulation can affect the eye through two mechanisms, either by damage to the extracellular matrix layer or interactions with receptor proteins. AGEs accumulate with age (Araki et al., 1992; Schleicher et al., 1997). Accumulation of AGEs in the eye would leads to abnormal crosslinking of extracellular matrix proteins, increase vascular stiffness, leading to changes in vascular structure and function. As a result, damage and leakage in the blood vessels and hyperproliferation can occur in diabetic retinopathy and AMD (Vlassara et al., 1994). AGEs formation is enhanced in hyperglycemic conditions in diabetic patients and reflects the severity of diabetic complications (Ono et al., 1998). Localization of AGEs in retinal blood vessels has been observed in patients with type 2 diabetes (Stitt et al., 1997). AGEs-induce oxidative stress cause consequent apoptosis of retinal pericytes (Bergers and Song, 2005). Pericytes play an important role in the maintenance of microvascular homeostasis. Loss of pericytes leads to angiogenesis, thrombogenesis, and endothelial cell injury, thus leading to diabetic retinopathy development (Eilken et al., 2017). In addition, AGEs interact with RAGE, resulting in increased intracellular production of free radicals and oxidative stress, which is then phosphorylates by MAP kinase and activates and increases expression of NF-κβ controlled genes causing vasoconstriction, angiogenesis, increased adhesion molecule expression, and inducing a procoagulant state associated with AMD and diabetic retinopathy (Tong and Yao, 2006; Sun et al., 2017).

There has been growing evidence suggesting that thymoquinone may be attributed to reducing the levels of AGEs (MDA, 4HNE) in cells. Administration of thymoquinone (10 mg/kg) in AD models has shown to lower the MDA level (Zaher et al., 2019). Besides, the protective effect of thymoquinone through reduction of 4HNE was observed in the model of renal (Dera et al., 2020a) and pulmonary diseases (Dera et al., 2020b). Reduction of MDA was also accompanied by increased levels of SOD, catalase and reduced glutathione (GSH). The findings were supported by an *in vitro* study Alhebshi et al. (2013), which showed that thymoquinone administration restored the redox balance in cultured rat hippocampal and cortical neurons displaying Aβ aggregation, as seen in AD (Alhebshi et al., 2013). Several studies also showed the same results in various condition such as in the liver and kidney of the rat model (Hosseinian et al., 2017), spinal cord injury model (Chen et al., 2018), diabetic model (Desai et al., 2015), renal induced toxicity (Sener et al., 2016), and ischemic brain model (Xiao et al., 2016).

Blocking of the Maillard reaction has been shown to be beneficial in preventing AGEs formation. Thymoquinone acts on AGEs by blocking the attachment of sugar to proteins, thereby attenuating glycosylation and oxidative stress while preventing protein crosslinking in a dose dependent manner (Losso et al., 2011; Anwar et al., 2014). Several studies have been shown to reduce glycation in sugar-induced AGEs model following administration of thymoquinone (Losso et al., 2011; Mahmood et al., 2013; Zafar et al., 2013; Benvidi et al., 2018; Pandey et al., 2018). Mahmood et al. (2013) and Zafar et al. (2013) observed the effect of glucose in inducing AGEs in model of diabetic. Both studies showed thymoquinone’s ability in reducing AGEs formation measured through thiobarbituric assay and AGEs fluorescence read at 480 nm (Mahmood et al., 2013; Zafar et al., 2013). In cases of fructose and BSA glycation, Pandey et al. (2018) shows thymoquinone reduced browning effect, indication of glycation process during AGEs formation (Pandey et al., 2018). The group also looked into carbonyl content and glycation aggregation index, which is a part of late stage of AGEs formation process. Data showed thymoquinone can reduce glycation aggregation index.

In support of those study, Losso et al. (2011) assessed the potential inhibitory effect of thymoquinone against AGEs from serum samples of diabetic patients through the use of hemoglobin-δ-gluconolactone, human serum albumin-glucose and the *N*-acetyl-glycyl-lysine methyl ester-ribose assay. Their results suggested thymoquinone at a dose as low as 10mM was able to suppress 78% of AGEs in blood plasma (Losso et al., 2011). The effect of thymoquinone on AGEs was also linked to the binding of thymoquinone to arginine-409 of thymoquinone as observed in BSA-induced AGEs (Benvidi et al., 2018). In addition, reduction of AGEs formation was also postulated to be due to the ability of thymoquinone in preventing the inactivation of SOD, an enzyme responsible as a defense mechanism in prevention of glycation (Anwar et al., 2014; Benvidi et al., 2018). Incubation of SOD with glycating agent, glucose, methyglyoxyl or both, has been shown to increase structural changes by SOD, thus alter it defense mechanism. However, thymoquinone was shown to reverse the effect by reducing the amount of SOD structural changes, attenuate AGEs formation as well as reduced protein crosslinking (Anwar et al., 2014). These data show the important of thymoquinone as an anti-glycating agent that may be useful to tackle AGEs formation in the ocular disorder.

### Modulation of Apoptosis by Thymoquinone

Apoptosis is defined as a programmed cell death intend to remove unwanted or damaged beyond repairs cells in the body. Shrinkage of the cell, fragmentation into membrane-bound apoptotic bodies and rapid phagocytosis by neighboring cells are the common characteristic of apoptosis (Nickells and Zack, 1996). Under normal circumstances, apoptosis is important to prevent accumulation of unwanted cells and making a way for new young cells to grow. Dysregulation of apoptosis can cause health problems. In cancer for instance, apoptosis process is absent followed with uncontrolled growth of new cells. In addition, excessive apoptosis also can create degeneration conditions in the body as seen in AD and PD, where the brain lose some part that is not supposed to die.

In the eye, apoptosis is considered a mechanism of cell death in ocular diseases including glaucoma, retinitis pigmentosa, cataract, AMD and diabetic retinopathy. Apoptosis has been found precedes these disease progression (Charakidas et al., 2005; Feenstra et al., 2013). DNA fragmentation analysis using terminal digoxigenin-labeled dUTP nick end labeling (TUNEL) in cataract patient revealed that apoptosis in cataract patient is higher compared to non-cataract patient. 92.6% of the samples tested shown average of 21.7 apoptotic cells per mm2, or 0.0047% of overall cell population and only three apoptotic epithelial cells per mm2 was detected from the non-cataract samples (Charakidas et al., 2005). Analysis of apoptosis modulator, Bcl-2 and Bax would allow determination of the susceptibility of a cell to apoptosis. Increase in the expression of both these proteins during disease progression was observed in glaucoma (Zalewska et al., 2004), retinitis pigmentosa (Comitato et al., 2014), cataract (Osnes-Ringen et al., 2016), AMD (Dunaief et al., 2002), and diabetic retinopathy (Khalfaoui et al., 2010). These findings suggest the important of apoptosis in the ocular disease development and study on possible modulation of these important process may leads to a crucial opportunity to deliver treatment sooner and hence preserve vision.

Apart from the earlier mentioned protective capability of thymoquinone, it also serves a dual function in apoptosis. In cancer cells, thymoquinone has been shown to promote apoptosis. In bladder cancer, thymoquinone was showed to induce apoptosis by enhancing the expression of the anti-apoptotic protein Bcl-2, blocking the release of cytochrome c and enhance the translocation of Bax from the cytoplasm to mitochondria (Zhang et al., 2018). Similar reaction was observed in breast cancer. Proapoptotic capacity of thymoquinone was observed through inhibition of anti-apoptotic genes, such as XIAP, survivin, Bcl-xL and Bcl-2, and increase in DNA fragmentation (Woo et al., 2013). Further study showed that pro-apoptotic activity of thymoquinone is followed with reduction in telomerase activity, an enzyme responsible for cellular resistance to apoptosis (Gurung et al., 2010). Thus, inhibition of telomerase by thymoquinone may induce apoptosis which is essential to prevent cancer.

On contrary, thymoquinone has been found to regulate apoptosis by reducing DNA fragmentation, downregulating of Bax and upregulating the Bcl-2 expression as well as decreasing downstream caspases 8, 9, and 3 in model of hepatic ischemia reperfusion injury, hence blocking apoptosis (Abd El-Ghany et al., 2009). In an *in vitro* model of AD, thymoquinone was shown to prevent apoptosis by blocking αβ-induced neurotoxicity (Alhibshi et al., 2019). Treatment with αβ_1–42_ for 48 h showed a significant decreased in cell viability to 63.5% as compared to the control group and cotreatment with 100 nM of thymoquinone restored the cell viability to 90%. The restoration of cell viability by thymoquinone was accompanied with significant reduction in ROS level to 2.6-fold as compared to sixfold in αβ_1–42_ only treatment. Ismail et al. (2013), also observed similar results with additional preserved intact cell bodies, extensive neurite networks, a loss of condensed chromatin and less free radical generation in primary cultured cerebellar granule neurons than those exposed to Aβ1–40 alone (Ismail et al., 2013). From these data, it is shown the selective capability of thymoquinone in inducing or inhibiting apoptosis in cells. This raises another interesting topic in studying the mechanism of thymoquinone in cells and applied it to ocular research.

### Effects of Thymoquinone on Mitochondrial Damage

Apoptosis is closely related to mitochondrial dysfunction. Mitochondrial dysfunction is described as a decrease in electron transport chain efficiency and a decrease in the synthesis of high-energy molecules like adenosine-5′-triphosphate (ATP). Mitochondrial dysfunction is a common feature in aging, and it also occurs in chronic diseases such as AD, muscular dystrophy, diabetes and cancer. It is important to note that ROS is primarily produced in the mitochondria, where they inhibit the mitochondrial electron transport chain’s complex I and reduce membrane integrity, resulting in higher ROS production (He et al., 2008). Mitochondrial-related mutation in the eye was first observed in Leber hereditary optic neuropathy (LHON). Mitochondrial DNA mutation in LHON affects the complex I of the electron transport chain, altering ATP synthesis. The resulting retinal ganglion cell apoptosis and optic nerve injury subsequently leads to blindness (Ghelli et al., 2003).

The mitochondria in retinal ganglion cells generate a large amount of energy required for nerve function in the unmyelinated ganglion cell axons. Glaucoma, like LHON, is an optic nerve disease characterized by accelerated apoptosis of retinal ganglion cells and their axons. It has been hypothesized that during the onset of glaucoma, an imbalance in the retinal ganglion cell axon energy requirement is caused by a change in blood flow dynamics in the optic nerve head, rendering the ganglion cells vulnerable to additional insults such as light entering the eye, which can further affect ganglion cell axon mitochondrial activity, and molecules released by astrocytes. If the mitochondria are unable to maintain normal function in these conditions, ganglion cell death can occur. Loss of retinal ganglion cells in glaucoma mouse models has been documented and this loss is due to the reduction in quantity of mitochondria and impairment of the mitochondrial function (Kim et al., 2015; Takihara et al., 2015).

More recently, the role of mitochondrial damage was investigated in diabetic retinopathy and AMD. An important cause of these conditions is an excess of pro-oxidants induced by the overproduction of ROS by the mitochondria throughout the retina. Increased retinal mitochondrial damage and disrupted mitochondrial membrane were observed in diabetic mouse models, together with elevated levels of ROS (Kanwar et al., 2007). In addition, in a diabetic rat model, the retinal mitochondria were shown to be leaky with diabetes, hence cytochrome c began to accumulate in the cytosol of retinal cells, observed *in vitro* rat models (Kowluru and Abbas, 2003). The RPE contains a high density of mitochondria to produce sufficient ATP to perform all its physiological processes. As a result, age-related mitochondrial dysfunction will lead to an increase in oxidative stress in the RPE, resulting in AMD. Wang et al. (2008) showed that mitochondrial damage was increased with age in the RPE of mice and rats (Wang et al., 2008). They concluded that the increase in mitochondrial damage in aging was due to a decrease in the cell’s DNA repair capability. In the case of AMD, Ferrington et al. (2017) and Golestaneh et al. (2017) investigated the mitochondrial function in RPE of AMD and healthy donor RPE. Their results showed reduction in mitochondrial function in RPE of AMD patients as compared to healthy donors (Ferrington et al., 2017; Golestaneh et al., 2017). It is also important to note, mitochondrial dysfunction can result in further increase in oxidative stress and thus leads to RPE functional decline and apoptosis. Taking these data together, maintaining the mitochondrial function and integrity is important to reduce the damage to the retinal ganglion cell and RPE cells in order to prevent blindness.

Like in the retina, the energy requirement in the brain is high to maintain its physiological function, thus mitochondrial dysfunction may pose a threat and lead to neurodegeneration as seen in AD and PD (Picone et al., 2014). The effect of thymoquinone on arsenic induced hippocampal toxicity and mitochondrial dysfunction in Wistar rats was studied (Firdaus et al., 2018). Arsenic is a compound found in pollutants and was found to induce oxidative stress as well as interruption in mitochondrial functions. Exposure to arsenic caused significant increase in intracellular ROS generation, mitochondrial dysfunction and apoptotic events. Administration of thymoquinone (2.5 and 5 mg/kg) was shown to inhibit the mitochondrial dysfunction by reducing the mitochondrial membrane potential in the rats hippocampal. Administration of thymoquinone concentration of 30 mg/kg for 10 consecutive days was shown to prevent mitochondrial dysfunction in liver of warm ischemia-reperfusion (I/R) injury rat model (Bouhlel et al., 2017, 2018). Thymoquinone was shown to also lessen the release of cytochrome C, an activator for caspase 9 and caspase 3 apoptotic pathways and thus preserve the mitochondrial function and prevent cell apoptosis. The prevention of liver mitochondrial damage could also be explained by the antioxidant and free radical scavenging activities of thymoquinone which in turn stabilized mitochondrial membrane permeability. Khan et al. (2012) showed the protective effect of thymoquinone against mitochondrial dysfunction in an *in vitro* model of AD using differentiated PC 12 cell line (Khan et al., 2012). In their study, thymoquinone pretreatment (1, 2, 4 μm) significantly counteracted the decreased in mitochondrial membrane potential. This showed an important mechanism of thymoquinone in combating mitochondrial dysfunction in cells. In addition, administration of 10 mg/kg of thymoquinone was shown to increase ATP synthesis in the renal cortex of kidney disease models (Dera et al., 2020a). These data together show a promising use of thymoquinone in maintaining retinal mitochondrial integrity and functions to prevent ocular neurodegeneration.

### Effect of Thymoquinone on the NF-κβ Signaling Pathway

The cells undergo cell-to-cell communication through signaling pathways. Various signaling pathways have been identified and associated with different diseases in the eye. Among all, NF-κβB is widely studied due to its important role in regulating key cellular processes such as inflammation, apoptosis, stress response, wound healing, and angiogenesis (Morgan and Liu, 2011). NF-κβ is a transcription factor present ubiquitously in the cell cytoplasm as a dimer bound to an inhibitory complex, Iκβ. NF- κβ molecule activation is triggered by a variety of stimuli including growth factors, cytokines, bacterial lipopolysaccharides, UV, and oxidative stress. Activation and translocation of NF-κβ molecules involved a series of phosphorylation and ubiquitination processes leading to gene transcription through binding of the NF-κβ dimer to the κβ sites of DNA sequences. Binding of NF-κβ dimer to the κβ sites of DNA sequences NF-κβ will induce gene transcription of specific proteins including various cytokines (Lingappan, 2018). NF-κβ is thought to be cell specific and it can even exert a dual function, for cell survival and induce cell death.

In the eye, the NF-κβ signaling is activated by retinal degeneration (Wu et al., 2002; Zeng et al., 2008). Zeng et al. (2008) studied the alteration of NF-κβ activity during retinal degeneration in *rd* mice (Zeng et al., 2008). Increase of phosphorylated NF-κB P65 proteins and augmented DNA binding activity were observed in *rd* mice with retinal degeneration. Wu et al. (2002) also demonstrated intense light exposure activates NF-κB in the photoreceptor cells of *rd* mouse model followed with increase in NF-κβ DNA–binding activity, and increased in expression of mRNA of Iκβα, a target gene of NF-κβ (Wu et al., 2002). Since NF-κβ activation resulted in the production of proinflammatory cytokines such as TNF-a and IL-1, which mediate inflammatory and immune responses, these findings may indicate that altering NF-κβ activity promotes photoreceptor apoptosis in the *rd* retina by initiating and perpetuating chronic inflammation. This hypothesis is supported by an *in vitro* study, where 60–70% of the 661W photoreceptor cells were found to undergo apoptosis with light exposure and level of P65 proteins were downregulated (Yang et al., 2007). Pretreatment with minocycline or sulforaphane countered the downregulation of P65 protein and as a result, photoreceptor apoptosis was inhibited. These data highlight NF-κβ as an extremely attractive target for therapeutic intervention in retinal degeneration.

Thymoquinone was shown to reduce activation of NF-κβ in cells. In one of the studies, BV2 microglial cells were stimulated with LPS after thymoquinone incubation. LPS-induced NF-κβ activation was studied by measuring the secretion of TNFα and other NF- κβ related inflammatory cytokines such as IL-6 and IL-1β (Nagi et al., 1999; Wang Y. et al., 2015; Velagapudi et al., 2017a). The levels of these NF- κβ related cytokines were significantly decreased. In addition, exposure of LPS on rat basophil cell lines showed a marked increase in TNFα production, and thymoquinone was able to reverse this effect at a concentration as low as 10 μM concentration (El Gazzar et al., 2007). Similar findings were noted on studies of thymoquinone on NF-κβ signaling and TNFα cytokines release in the rat’s liver (Sayed and Morcos, 2007).

Thymoquinone (10, 15, 20, 25 μmol/L) suppressed NF-κβ activation in dose dependent manner, specifically by suppression direct binding of nuclear p65 and recombinant p65 to the DNA in 0.1 nmol/L TNFα and LPS stimulated cells. To confirm the finding, cells were transfected with p65 plasmid containing cysteine residue 38 mutated to serine and the binding inhibition by thymoquinone were flattened. Their data showed that thymoquinone suppressed NF-κβ activation correlated with sequential inhibition of the activation of IKK, IKβα phosphorylation, IKβα degradation, p65 phosphorylation, p65 nuclear translocation, and the NF-κβ-dependent reporter gene expression. Expression of NF-κβ regulated antiapoptotic genes (IAP1, IAP2, XIAP Bcl-2, Bcl-xL, and survivin), proliferative (cyclin D1, cyclooxygenase-2, and c-Myc), and angiogenic (matrix metalloproteinase-9 and vascular endothelial growth factor) gene products were also downregulated with thymoquinone treatment (Sethi et al., 2008). Similar findings were reported by Velagapudi et al. (2017b).

Wang Y. et al. (2015) and Velagapudi et al. (2017b) suggested that alteration of NF- κβ by thymoquinone may also be mediated through inhibition of Iκβα phosphorylation or P65 phosphorylation (Wang Y. et al., 2015; Velagapudi et al., 2017b). Thymoquinone has also been reported to exert inhibitory effects on AGE-induced NF-κB-activation in human proximal tubular epithelial cells (pTECs) stimulated with advanced glycation end products (AGEs) (Sayed and Morcos, 2007). All these findings showed the mechanism of thymoquinone in reducing the pro-inflammatory NF-κβ signaling pathways, which can be a reflection of its potential neuroprotective effects in ocular degeneration.

### Effect of Thymoquinone on Nrf2 Signaling Pathway

Apart from NF-κβ signaling, thymoquinone also affects – Nrf2 signaling, a master regulator of oxidative stress. Both NF-kB and Nrf2 signaling pathways are inter-related. Increase in NF-κB activation is followed by a decline in Nrf2 signaling pathway, thus further increasing inflammation response. Nrf2 is a transcription factor that is involved in the cellular defense against oxidative stress by controlling the expression of antioxidant-related gene expression resulting in anti-inflammatory response in the cells (Vomund et al., 2017). Nrf2 is thought to play a pivotal role in inflammation because its protein products are responsible for detoxification and elimination of oxidative stress in the cells (Nguyen et al., 2009). Mounting evidence suggests that aging induces a decline in the antioxidant capacity via a reduction in Nrf2 signaling.

The importance of Nrf2 signaling in the pathogenesis of AMD was highlighted when Nrf2-deficient mice developed features similar to human AMD. These mice exhibit pathological characteristics of human AMD including progressive RPE and Bruch’s membrane degeneration, drusen deposits and lipofuscin accumulation, and decreased electroretinography responses (Zhao et al., 2011).

In the RPE, under oxidative stress conditions, the Nrf2 signaling is impaired. Nrf2 transcription factors are lowered, and hence are not able to translocate to the nucleus to induce an antioxidant response. This renders the RPE and photoreceptors vulnerable to damage. Catanzaro et al. (2020) studied the role of Nrf2 on wild type (WT) and Nrf2-silenced (siNrf2)- ARPE-19 cells exposed to various AMD-related noxae (H2O2, 4-HNE, MG132 + Bafilomycin) (Catanzaro et al., 2020). The study showed that in Nrf2 knockout group, cells present a higher susceptibility to oxidative insults as compared to WT groups, suggesting that the Nrf2 induction represents an efficient defensive strategy to prevent the stress-induced damage. Increasing Nrf2-regulated glutathione levels and NAD(P)H attenuated ultraviolet light-induced RPE damage by Gao and Talalay (2004). Carotenoids zeaxanthin and lutein were shown to preserve photoreceptors against light damage by mitigating oxidative stress through Nrf2 signaling (Frede et al., 2017; Yu et al., 2018).

Thymoquinone has been shown to increase activation of Nrf2 signaling. Anti-inflammatory effects of thymoquinone in BV2 microglial cells occurred via the Nrf2 signaling pathway. Activation of Nrf2 signaling by thymoquinone was found to be accompanied by increased nuclear localization, DNA binding and transcriptional activity of Nrf2, as well as increasing protein levels of HO-1 and NQO1. Suppression of Nrf2 activity through siRNA or with the use of trigonelline resulted in the loss of this effect (Velagapudi et al., 2017b). The same results were also observed in models of lung fibrosis (Ahmad et al., 2020), kidney injury (Al Fayi et al., 2020), lung injury (Mao et al., 2020), and allergy response (Dera et al., 2020c). Taken together, these data demonstrated the anti-inflammatory effect of thymoquinone through Nrf2 signaling.

### Modulation of Cellular Proteostasis by Thymoquinone

Proteostasis is regulated by a complex network of cellular mechanisms that monitors the concentration, folding, cellular localization, and interactions of proteins from their synthesis through their cellular degradation. Decline in proteostasis is one of the hallmarks of aging and accumulation of damaged protein leads to age related diseases such AD, PD, AMD, glaucoma and cataract. Proteosomes complex is a non-lysosomal ATP-dependent degradation system responsible in removal of damaged, oxidized and misfolded proteins in cells through a process called autophagy (Saez and Vilchez, 2014).

In the eye, autophagy occurs in the lens, cornea, photoreceptors, and RPE cells. Autophagy plays a role in lens fiber cells and corneal maturation (Costello et al., 2013). And maintains the inner segment turnover and survival of the photoreceptor. Within RPE cells, autophagy is involved in aspects of development, cell survival in response to stress, melanin degradation, as well as the degradation of toxic cellular components or damaged organelles. The importance of autophagy in these tissue has been discussed (Frost et al., 2014).

Damaged protein degradation is crucial in cells before the accumulated proteins aggregated. The 20S proteasome is the central core of this system and acts as a part of the intracellular antioxidant defense system (Bonfili et al., 2008). It is estimated that more than 20% of proteins in mammalian cells are degraded by the 20S proteasomes (Baugh et al., 2009). The 20S proteasomes recognized damage proteins through specific degradation signals such as multi-ubiquitin chain tag (Sahu et al., 2021). For a protein to be tagged for degradation, a series of reaction was carried out by E1 (ubiquitin-activating), E2 (ubiquitin-conjugating), and E3 (ubiquitin-ligating) enzymes to create a ubiquitin tag that consist of at least 4 ubiquitin molecules. The chain of ubiquitin directs the damaged proteins to the 20S proteasomes where proteolytic degradation occurs (Ravid and Hochstrasser, 2008; Saez and Vilchez, 2014).

Thymoquinone has been demonstrated to induces selective and time-dependent proteasome complex modulation. However, the mechanism of thymoquinone has only been observed in cancer cells (Cecarini et al., 2010). In glioblastoma cells, thymoquinone has been shown to induce proteosome complex inhibition. This inhibition is due to compromised 20S proteosomes complex functionality and not to downregulation of its synthesis as observed through western blotting and 20S proteosomes activity analysis. In addition, E3 ubiquitin ligase was also downregulated with thymoquinone treatment in acute leukemia model (Alghamdi et al., 2020). Based on this evidence in cancer subject and with the importance of autophagy in the eye, study on thymoquinone in proteosome might provide a novel mechanism of thymoquinone in ocular disease application.

## Conclusion

Thymoquinone has been traditionally and still widely used to treat various conditions due to its medicinal properties. Modern scientific studies showed the beneficial neuroprotective effects of thymoquinone on AD and PD experimental models. Further studies demonstrated the mechanistic pathways involved in the anti-inflammatory and antioxidant properties of thymoquinone in various *in vitro* and *in vivo* models. Thymoquinone has been shown to exert its protective effects through the NF-κβ and Nrf2 signaling pathways, and the anti-oxidative activity. These findings provide the basis of the potential therapeutic effects of thymoquinone in ocular degenerative diseases as similar mechanisms of action occur and signaling pathways are involved in degenerative retinal conditions such as AMD. More studies of thymoquinone effects on the retina are needed to provide a deeper understanding of the mechanism of thymoquinone on a cellular level, before clinical trials can be performed.

## Author Contributions

NM drafted the manuscript. TK, NM, and LP reviewed and revised the initial manuscript. NK reviewed the manuscript draft. All authors read and approved the final manuscript.

## Conflict of Interest

The authors declare that the research was conducted in the absence of any commercial or financial relationships that could be construed as a potential conflict of interest.

## Publisher’s Note

All claims expressed in this article are solely those of the authors and do not necessarily represent those of their affiliated organizations, or those of the publisher, the editors and the reviewers. Any product that may be evaluated in this article, or claim that may be made by its manufacturer, is not guaranteed or endorsed by the publisher.
